# Vitamin D Status and Virologic Response to HCV Therapy in the HALT-C and VIRAHEP-C Trials

**DOI:** 10.1371/journal.pone.0166036

**Published:** 2016-11-10

**Authors:** Erikka Loftfield, Thomas R. O’Brien, Ruth M. Pfeiffer, Charles D. Howell, Ron Horst, Ludmila Prokunina-Olsson, Stephanie J. Weinstein, Demetrius Albanes, Timothy R. Morgan, Neal D. Freedman

**Affiliations:** 1 Division of Cancer Epidemiology and Genetics, National Cancer Institute, National Institutes of Health, Bethesda, MD, United States of America; 2 Department of Medicine, Howard University College of Medicine, Washington, DC, United States of America; 3 Heartland Assays, Ames, IA, United States of America; 4 VA Long Beach Healthcare System, Long Beach, CA, United States of America; Taipei Veterans General Hospital, TAIWAN

## Abstract

More than 150 million people worldwide are chronically infected with hepatitis C virus (HCV) and face higher risk of cirrhosis and hepatocellular carcinoma. Highly effective HCV treatments have recently been developed; however, they are costly and therefore poorly suited for application in resource-limited settings where HCV burden is high. Pegylated-interferon alpha (PEG-IFNα) and ribavirin (RBV) therapy is far less costly, but also less effective. Vitamin D supplementation has been proposed as an inexpensive adjuvant to treatment, however, prior epidemiological evidence on its effectiveness is inconsistent, with little data available among African Americans who naturally have lower vitamin D concentrations. We thus evaluated associations between baseline vitamin D status, measured by circulating 25-hydroxyvitamin D (25(OH)D), which is considered to be the best marker of vitamin D status in humans, and subsequent response to PEG-IFNα/RBV therapy in two large clinical trials that together included 1292 patients infected with HCV genotype 1. We used race-stratified logistic regression models to evaluate multivariable-adjusted associations of 25(OH)D with early virologic response (EVR; 2-log_10_ HCV RNA decline at week 12) and sustained virologic response (SVR). Among African Americans, we saw no associations. Among European Americans, we saw no association with low vitamin D (≤20 ng/mL) versus sufficient concentrations (20-<30 ng/mL). However, patients with 25(OH)D ≥30 ng/mL were actually less likely to attain EVR (OR = 0.64, 95% CI = 0.43–0.94) than those with sufficient concentrations, with a similar but non-significant association observed for SVR (OR = 0.49, 95% CI = 0.20–1.17).

**Conclusion:**

Higher vitamin D status was not beneficially associated with responses to therapy; if anything, patients with higher vitamin D concentrations were less likely to attain SVR. Our data do not support a role for vitamin D supplementation as an adjuvant therapy for HCV.

## Introduction

Worldwide 130 to 170 million people, or 2 to 3% of the global population, are chronically infected with hepatitis C virus (HCV) [1, 2]. In the absence of treatment, chronic HCV infection substantially elevates risk for cirrhosis and hepatocellular carcinoma [3, 4], and an estimated 500,000 people die annually from HCV-related liver diseases [5]. Pegylated-interferon alpha and ribavirin (IFNα/RBV) combination therapy results in sustained virologic response (SVR) in 40–50% of treatment naïve patients infected with HCV genotype 1 [6, 7], which accounts for ≥70% of HCV infections in the United States [8] and 90% of HCV infections among African Americans [9]. Direct-acting antiviral agents (DAAs) are far more effective for treating HCV genotype 1[10], but are also far more expensive than PEG-IFNα/RBV therapy, costing upwards of $84,000/patient [11, 12]. Cost is one of the greatest barriers to HCV treatment [13], especially in developing regions, including Africa and Asia, where the burden of HCV infection is highest [1, 14, 15], [16]. Predictors of poor virologic response to PEG-IFNα/RBV therapy include African ancestry [9, 17] and *IFNL4* genotype [18–20], and a recent clinical trial implicated these factors as predictive of lower response rates to a commonly used DAA regimen (ledipasvir and sofosbuvir) as well [21].

Results from supplementation and observational studies suggest that vitamin D deficiency may adversely impact virologic response to PEG-IFNα/RBV therapy [22–24]. Vitamin D binds and activates the vitamin D receptor (VDR); VDR is a potent transcriptional regulator active in both the innate and adaptive immune system, including the interferon pathway, which is essential in HCV clearance [25, 26]. Additionally, vitamin D has been shown to inhibit HCV replication in cell culture [27–29]. However, prior findings on the relationship between vitamin D and response to HCV therapy have been inconsistent. Highlighting this lack of consensus, two recently published meta-analyses of the association of vitamin D status with SVR to PEG-IFNα/RBV therapy came to different conclusions [30, 31]. Prior studies have often been unable to adjust for potential confounding factors such as *IFNL4* genotype, liver fibrosis stage, body mass index (BMI) and season of blood draw, and few studies have examined associations among individuals of African ancestry, who naturally have low vitamin D concentrations [32]. Finally, data on the temporal relationship between vitamin D and HCV are lacking [30, 31].

Here we conducted an observational study using data from two clinical trials with high quality patient data on known predictors of virologic response, Hepatitis C Antiviral Long-Term Treatment against Cirrhosis (HALT-C) and Viral Resistance to Antiviral Therapy of Chronic Hepatitis C (VIRAHEP-C), to assess whether pre-treatment vitamin D concentrations are independently associated with virologic response to PEG-IFNα/RBV among European American and African American patients.

## Materials and Methods

### HALT-C Study Population and Study Design

The design of the HALT-C trial (ClinicalTrials.gov Identifier: NCT00006164) has been described elsewhere [33, 34]. Briefly, 1145 HCV-positive patients who had failed previous interferon therapy and had an Ishak fibrosis score ≥3, but no history of hepatic decompensation, hepatocellular carcinoma, uncontrolled medical or psychiatric conditions, or contraindications to interferon treatment were enrolled in the lead-in phase of this study between August 2000 and August 2004 from 10 study centers in the United States. During the lead-in phase, patients received 180 μg of PEG-IFNα weekly and either 1000 or 1200 mg of oral RBV daily according to body weight of <75 kg or ≥75 kg, respectively. Patients with undetectable serum HCV RNA at week 20 continued therapy for a total 48 weeks, barring viral breakthrough or relapse, and were assessed for SVR 24 weeks after treatment was completed [34, 35]. Our study included all lead-in phase HALT-C participants of European and African American ancestry (n = 126 excluded) who were infected with HCV genotype 1 (n = 99 excluded) and had an available baseline blood sample for vitamin D measurement (n = 9 excluded); the final sample size was N = 911 (n = 166 African American; n = 745 European American).

### VIRAHEP-C Study Population and Study Design

The design of the VIRAHEP-C Study (ClinicalTrials.gov Identifier: NCT00038974) has been described elsewhere [17]. Briefly, this study enrolled 401 treatment naïve patients with chronic HCV genotype 1 infection from eight study centers in the United States between July 2002 and December 2003 who identified themselves as “African American/black” or “Caucasian/white” and not as “both” or “other” [17]. Patients received 180 μg of PEG-IFNα weekly and either 1000 or 1200 mg of oral RBV daily according to body weight of <75 kg or ≥75 kg, respectively. Patients with detectable HCV RNA levels at week 24 were considered nonresponders and therapy was stopped. Patients with undetectable HCV RNA levels at week 24 continued therapy for another 24 weeks and were assessed for SVR 24 weeks after treatment was completed [17]. For the current analysis, we restricted the VIRAHEP-C population to those with available baseline serum for measuring vitamin D (n = 20 excluded). The final VIRAHEP-C analytic sample size was N = 381 (n = 184 African American; n = 197 European American).

### Assessment of Virologic Response to Treatment

For patients enrolled in HALT-C, serum samples were tested with quantitative reverse-transcriptase polymerase chain reaction (qRT-PCR) at the University of Washington Virology Laboratory (Seattle, Washington) using the quantitative Roche COBAS Amplicor HCV Monitor Test, v.2.0 assay (sensitivity 600 IU/mL) and, if negative, using the Roche COBAS Amplicor HCV Test, v.2.0 assay (sensitivity 100 IU/mL) (Roche Molecular Systems, Branchburg, New Jersey) [35, 36]. HCV genotyping was determined with the INNO-LiPA HCV II kit (Siemens Medical Solutions Diagnostics, Tarrytown, New York).

For those enrolled in VIRAHEP-C, serum samples were tested at the SeraCare BioServices Laboratory (Gaithersburg, Maryland) using the quantitative Roche COBAS Amplicor HCV Monitor Test, v.2.0 assay (sensitivity 600 IU/mL) and, if negative using the Amplicor assay (sensitivity 50 IU/mL) (Roche Molecular Diagnostics, Alameda, CA). HCV genotyping was determined with the VERSANT HCV Genotype Assay (Bayer, Tarrytown, New York).

In both studies, serum HCV RNA level was measured at multiple time points including baseline, week 12 of treatment, end of treatment, and 24 weeks after treatment completion. In VIRAHEP-C serum HCV RNA level was also assessed at day 28 of treatment; prior studies have shown that this outcome is a strong predictor of SVR. In the main analyses, early virologic response (EVR) was defined as a ≥2-log_10_ decline in serum HCV RNA level at week 12 [37]; lack of EVR at week 12 has been found to accurately predict lack of SVR [6, 38]. In HALT-C, 38 participants had missing data on HCV RNA at week 12 and were thus excluded from the EVR analysis. In VIRAHEP-C only, day 28 change in HCV RNA level was defined as log_10_ (HCV RNA) _baseline_−log_10_ (HCV RNA) _day 28;_ 14 VIRAHEP-C participants had missing data on HCV RNA at treatment day 28 and were thus excluded from this analysis. SVR was defined for both studies as the absence of detectable HCV RNA 24 weeks after treatment completion.

### Laboratory Analyses

Baseline serum samples for 911 HALT-C patients and 381 VIRAHEP-C patients, as well as week 12 serum samples for 349 of the 381 VIRAHEP-C patients, were assayed at Heartland Assays, Inc. (Ames, Iowa). Measurement of 25(OH)D was conducted for each study by means of a direct, competitive chemiluminescence immunoassay using the DiaSorin LIAISON 25(OH)D TOTAL assay (DiaSorin, Inc., Stillwater, Minnesota), which is cospecific for 25-hydroxyvitamin D_3_ and 25-hydroxyvitamin D_2_ [39, 40]. For VIRAHEP-C patients with baseline and 12 week paired serum samples, aliquots were randomly ordered within batches. Quality control samples from a single pooled sample of patients from the HALT-C Study were randomly placed in each batch at a proportion of approximately 5%. Intra- and inter-assay coefficients of variation for blinded quality control samples were 6.5% and 2.8% in HALT-C and 5.1% and 3.6% in VIRAHEP-C, respectively.

As previously described, genotyping of *IFNL4*-ΔG/TT (rs368234815) was conducted in the Laboratory of Translational Genomics (NCI, Gaithersburg, Maryland) with a custom TaqMan allelic discrimination genotyping assay (Life Technologies, Foster City, California) [19, 20].

### Statistical Analyses

Vitamin D status was analyzed both as a continuous (ng/mL) and categorical variable. First, we tested for differences in the distribution of baseline 25(OH)D between early responders and nonresponders, stratified by study and race, using the Brown-Mood test [41]. For the primary analyses, categories of vitamin D were defined according to the Institute of Medicine (IOM) cutpoints: deficiency (<12 ng/mL); inadequacy (12 to <20 ng/mL); sufficiency (20 to <30 ng/mL); and no consistent evidence for added benefit but potential concern for harm (≥30 ng/mL) [32, 42]. We also performed analyses based on the 25(OH)D categories used in the meta-analysis by García-Álvarez et al. (<20 ng/mL versus ≥20 ng/mL) that found evidence for an association between vitamin D status and SVR. In secondary analyses, we considered race and season-specific quartiles of vitamin D since HALT-C and VIRAHEP-C studies included sizable African American populations and baseline serum draw dates occurred during both summer (June-November) and winter (December-May) seasons [39].

Because decline in HCV RNA at treatment day 28 has been strongly associated with *IFNL4* genotype [17], linear models were used to estimate unadjusted and multivariable-adjusted mean 28-day change in HCV RNA stratified by race and baseline 25(OH)D level. For binary outcomes, logistic regression models were also stratified by race, as African Americans are known to have both lower vitamin D concentrations and poorer response to pegylated IFNα/RBV treatment than European-American patients. We present study-specific and overall ORs from models adjusted for baseline age, sex, BMI, HCV RNA level, homeostatic model assessment of insulin resistance (HOMA) score, *IFNL4* genotype, and the markers of liver disease severity which independently predicted virologic response in each study (albumin, AST/ALT ratio, and platelet count in HALT-C; and AST/ALT ratio in VIRAHEP-C). Additionally, data on treatment site was available in VIRAHEP-C and was included in multivariable models but did not meaningfully impact OR estimates. Results were similar following adjustment for additional markers of liver disease severity (e.g. Ishak stage).

For multivariable-adjusted models, we used Imputation and Variance Estimation Software (IVEware) to perform multiple imputations of missing values. [43]; to combine these results and generate valid statistical inferences, we used the SAS procedure PROC MIANALYZE. Finally, we used a random effects meta-analysis approach to obtain overall ORs and 95% CIs for HALT-C and VIRAHEP-C using Stata software. Study heterogeneity was assessed with I^2^ [44].

To better understand the impact of liver disease and season of baseline blood draw on virologic response, we conducted the following secondary analyses: 1) examined associations separately among patients with cirrhosis, defined as Ishak stage 5 or 6, and those with fibrosis, defined as an Ishak stage 1 through 4; 2) stratified by season of baseline blood draw. To evaluate the longitudinal relationship between 25(OH)D and HCV RNA levels we calculated the change in 25(OH)D concentration for 349 patients in VIRAHEP-C with paired baseline and week 12 serum samples and estimated the mean change in 25(OH)D concentration stratified by EVR status and race. We used a paired t-test to assess whether there was a change in individual 25(OH)D concentration from baseline to week 12. Since 12-week change in 25(OH)D concentration was approximately normally distributed, we used an unpaired t-test to compare the mean change in 25(OH)D between responders and nonresponders to treatment at week 12.

All tests were two-sided, and a P-value<0.05 was considered statistically significant. Analyses were performed using SAS software (release 9.3, SAS Institute, Cary, North Carolina), IVEware (version 0.1, Survey Research Center, University of Michigan) and Stata software (version 14.0, StataCorp, College Station, Texas).

### Human Subjects

The HALT-C and VIRAHEP-C study protocols conformed to the ethical guidelines of the 1975 Declaration of Helsinki as reflected in *a priori* approval by the institutional review boards of the National Institute of Diabetes and Digestive and Kidney Diseases (NIDDK), the participating HALT-C trial sites (University of California-Irvine/VA Medical Center-Long Beach; University of Southern California; University of Colorado; University of Connecticut; NIDDK Liver Disease Section; Massachusetts General Hospital; University of Massachusetts; University of Michigan; Saint Louis University; University of Texas Southwestern; Virginia Commonwealth University) and the participating VIRAHEP-C trial sites (University of California, San Francisco; University of Miami School of Medicine; Rush University; University of Maryland School of Medicine; University of Michigan Medical Center; New York-Presbyterian Medical Center; University of North Carolina) and written consent was obtained from all patients.

## Results

### Patient Characteristics

Demographic and baseline clinical factors differed according to varying study enrollment criteria in the two trials (Table 1). For example, the HALT-C study was restricted to patients with advanced fibrosis or cirrhosis who had previously failed interferon treatment whereas those in the VIRAHEP-C study were treatment naïve. Accordingly, cirrhosis was much more prevalent in HALT-C than in VIRAHEP-C (36.4% vs 7.1%), and a lower proportion of HALT-C patients as compared with VIRAHEP-C attained SVR (13.3% vs 39.1%) (Table 1). VIRAHEP-C study enrolled approximately equal numbers of African and European Americans, but only 18.2% of the HALT-C patients were African American.

**Table 1:** Baseline characteristics by study.

	Study	
Baseline characteristic	HALT-C (n = 911)	VIRAHEP-C (n = 381)
Age (y), mean (SD)	50.0 (7.2)	48.2 (7.9)
Sex, n (%)		
Male	660 (72.4)	247 (64.8)
Female	251 (27.6)	134 (35.2)
Race, n (%)		
African American	166 (18.2)	184 (48.3)
European American	745 (81.8)	197 (51.7)
*IFNL4* (rs368234815) genotype, n (column %)		
TT/TT	171 (18.8)	92 (24.1)
ΔG/TT	448 (49.2)	152 (39.9)
ΔG/ΔG	195 (21.4)	89 (23.4)
Missing	97 (10.6)	48 (12.6)
Ishak stage, n (%)		
≤2	73 (8.0)	240 (63.0)
3 or 4	505 (55.4)	111 (29.1)
5 or 6	332 (36.4)	27 (7.1)
Missing	1 (0.1)	3 (0.8)
BMI (kg/m^2^), n (%)		
<18.5	1 (0.1)	
18.5 to <25	158 (17.3)	93 (24.4)
25 to <30	369 (40.5)	138 (36.2)
≥30	384 (42.2)	145 (38.1)
Missing	0 (0)	5 (1.3)
Season of baseline blood draw, n (%)		
Summer	452 (49.6)	230 (60.4)
Winter	459 (50.4)	151 (39.6)
SVR, n (%)		
Yes	121 (13.3)	149 (39.1)
No	790 (86.7)	232 (60.9)
25(OH)D (ng/mL), mean (SD)	21.5 (10.1)	21.9 (11.0)
European Americans	23.4 (9.8)	27.7 (11.)
African Americans	13.0 (6.1)	15.7 (6.7)
HCV RNA level log_10_ (IU/mL), mean (SD)	6.4 (0.5)	6.3 (0.7)
Albumin (g/dL), mean (SD) *	3.9 (0.4)	4.1 (0.4)
AST/ALT ratio, mean (SD)	0.85 (0.29)	0.81 (0.30)
Bilirubin (mg/dL), mean (SD) †	0.8 (0.4)	0.7 (0.4)
Platelet count (x10^3^/mm^3^), mean (SD) ‡	172 (65)	214 (67)

Abbreviations: 25(OH)D, 25-hydroxyvitamin D; AST/ALT, aspartate transaminase and alanine transaminase ratio; BMI, body mass index; HCV, hepatitis C virus; SD, standard deviation; SVR, sustained virologic response

* 31% of participants in VIRAHEP-C were missing data on albumin

† 1% of participants in VIRAHEP-C were missing data on bilirubin

‡ 2% of participants in VIRAHEP-C were missing data on platelet count

### Vitamin D Status in HALT-C and VIRAHEP-C

Despite the differences in the HALT-C and VIRAHEP-C study populations, vitamin D concentrations in the two studies were comparable. Overall, vitamin D deficiency (<12 ng/mL) was present in 17.2% of HALT-C participants and 19.4% of the VIRAHEP-C participants, and, in both the HALT-C and VIRAHEP-C studies, approximately 48% of patients had deficient or inadequate 25(OH)D concentrations, defined as <20 ng/mL, at baseline (S1 and S2 Tables). As expected, vitamin D status differed markedly by race. In HALT-C, vitamin D deficiency was found in 50.6% of African American patients compared to 9.8% of European Americans, and, in Virahep-C, those proportions were 33.7% and 6.1%, respectively (data not shown in table).

In both studies, lower concentrations of vitamin D were associated with most but not all markers of underlying liver disease, such as higher serum AST/ALT, higher measures of alkaline phosphatase and lower albumin (S1 and S2 Tables). In addition, vitamin D concentrations were inversely associated with Ishak fibrosis stage in HALT-C, although only when we compared vitamin D concentrations among patients with cirrhosis (Ishak 5–6) to those with fibrosis (Ishak 2–4), P-trend = 0.003; (more detailed comparisons are presented in S1 and S2 Tables).

### Vitamin D Status and Treatment Response

**Fig 1:**
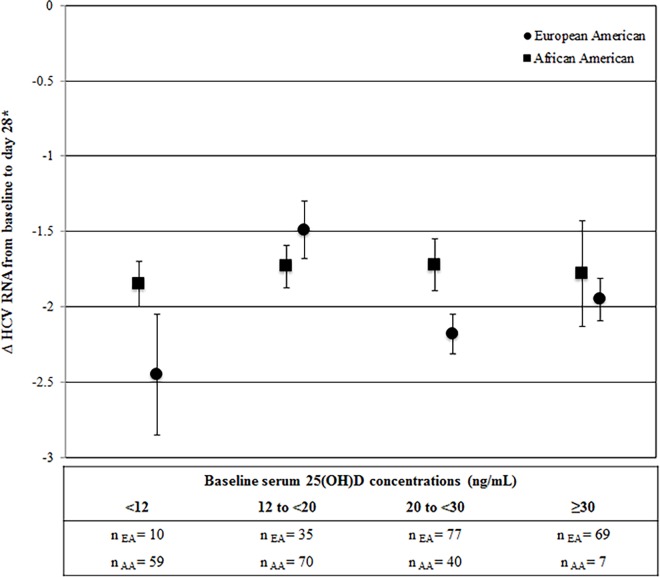
Adjusted mean ΔHCV RNA level (SE) from baseline to day 28 of PEG-IFNα/RBV treatment by baseline serum 25(OH)D status among European and African Americans in the VIRAHEP-C study (n = 191). Mean ΔHCV RNA level was calculated as the change in log_10_ transformed viral load from baseline to day 28 of treatment [i.e. log_10_ (HCV RNA) _day 28_– log_10_ (HCV RNA) _baseline_] and adjusted for age (years), sex, IFNL4 genotype (ΔG/ΔG, ΔG/TT, TT/TT), BMI (kg/m^2^), baseline HCV RNA level (log_10_ transformed IU/mL), HOMA score, treatment site, and AST/ALT. Figure abbreviations: 25(OH)D, 25-hydroxyvitamin D; AA, African American; AST/ALT, aspartate transaminase and alanine transaminase ratio; BMI, body mass index; EA, European American; HCV, hepatitis C virus; HOMA, homeostasis model assessment; IOM, Institute of Medicine; LS, least squares; PEG-IFNα/RBV, pegylated interferon alpha and ribavirin; SE, standard error.

In VIRAHEP-C, HCV RNA levels were examined during the first 28 days of treatment (Fig 1 and S3 Table). Among African American participants, we saw a similar decrease in HCV RNA from baseline to day 28 (~1.8 log_10_ IU/Ml) with treatment across the four 25(OH)D categories. In European American participants, we did not observe a consistent relationship between 25(OH)D category and decrease in viral levels, as there were greater declines in HCV RNA among participants in the <12 and the 20 to <30 categories of vitamin D than for the intermediate category (12 to <20).

Early response to PEG-IFNα/RBV therapy, defined as ≥2-log_10_ decline in HCV RNA level from baseline to week 12, was achieved by 48% and 53% of patients in HALT-C and VIRAHEP-C, respectively. In both studies, EVR was associated with several previously established factors (European American ancestry, *IFNL4*-TT/TT genotype, lower Ishak fibrosis stage, lower HOMA or HOMA2 scores, lower measures of alkaline phosphatase, higher serum AST/ALT ratio, and higher platelet counts [data not shown]); however, higher baseline 25(OH)D was not associated with EVR (Table 2) or SVR (data not shown).

**Table 2:** Serum 25(OH)D concentrations (ng/mL) by early virologic response (≥ 2-log_10_ decline in HCV RNA level from baseline to week 12).

Study	Race	Early virologic response				P*
		Yes		No		
		n	Median (IQR)	n	Median (IQR)	
HALT-C †	European Americans	373	21.9 (16.7–27.4)	341	23.0 (16.2–30.0)	0.261
	African Americans	48	12.5 (8.9–17.1)	111	11.5 (8.4–16.4)	0.456
VIRAHEP-C ‡	European Americans	126	27.0 (21.1–34.4)	71	25.9 (18.8–36.2)	0.696
	African Americans	76	13.7 (11.0–21.1)	108	15.1 (10.7–20.1)	0.370

* P-value for Brown-Mood test, two-sided normal approximation

† n = 38 HALT-C participants did not have HCV RNA levels measured at a week 12 and were thus excluded from the EVR analysis

Abbreviations: 25(OH)D, 25-hydroxyvitamin D; HCV, hepatitis C virus; IQR, interquartile range

Next, we evaluated the associations of vitamin D status with EVR and SVR. Among European Americans in both studies, a lower proportion of those attaining SVR had high baseline 25(OH)D, ≥30 ng/mL, as compared with those who failed treatment (9% vs. 22% and 33% vs. 38%, respectively) (data not shown in table). These associations persisted among European Americans in multivariable adjusted models, with comparable overall odds ratios for high vitamin D status, ≥30 ng/mL, as compared with sufficient vitamin D status, 20 to <30 ng/mL observed for EVR (OR = 0.64, 95% CI = 0.43–0.94) and SVR (OR = 0.49, 95% CI = 0.20–1.17), although there was significant heterogeneity between the two studies for SVR (HALT-C: OR = 0.31, 95% CI = 0.14–0.66; VIRAHEP-C: OR = 0.75, 95% CI = 0.37–1.55) and the overall association did not reach statistical significance (Table 3). We found no evidence of a dose-response relationship between vitamin D concentrations and rapid viral response at day 28 in VIRAHEP-C or SVR in either study (all P-trend>0.1; data not shown). For comparison with the meta-analysis by García-Álvarez et al., we also considered a cutpoint of 20 ng/mL, but were unable to replicate the reported association between vitamin D status and SVR (Table 3).

**Table 3:** Associations of baseline serum 25(OH)D status with response to PEG-IFNα/RBV treatment for HCV among European Americans.

	Baseline serum 25(OH)D concentrations (ng/mL)					
	IOM cut points				Meta-analysis cut points (31)	
	<12	12 to <20	20 to <30 (Ref)	≥30	<20	≥20 (Ref)
**Early virologic response**						
HALT-C, n responders/n total *	33/70	114/206	167/295	59/143	147/276	226/438
Multivariable adjusted OR (95% CI) †	0.96 (0.52–1.74)	1.08 (0.72–1.62)	1.00	0.60 (0.38–0.95)	1.24 (0.88–1.74)	1.00
VIRAHEP-C, n responders/n total	7/12	18/36	57/79	44/70	25/48	101/149
Multivariable adjusted OR (95% CI) ‡	0.47 (0.10–2.18)	0.23 (0.08–0.64)	1.00	0.75 (0.33–1.72)	0.31 (0.13–0.73)	1.00
Overall OR (95% CI) §	0.87 (0.50–1.52)	0.53 (0.12–2.45) ^║^	1.00	0.64 (0.43–0.94)	0.66 (0.17–2.53) ^║^	1.00
**Sustained virologic response**						
HALT-C, n responders/n total	7/73	34/221	59/306	10/145	41/294	69/451
Multivariable adjusted OR (95% CI) †	0.57 (0.23–1.38)	0.80 (0.47–1.36)	1.00	0.31 (0.14–0.66)	0.99 (0.62–1.58)	1.00
VIRAHEP-C, n responders/n total	7/12	13/36	46/79	33/70	20/48	79/149
Multivariable adjusted OR (95% CI) ‡	1.16 (0.29–4.64)	0.36 (0.15–0.91)	1.00	0.75 (0.37–1.55)	0.55 (0.26–1.17)	1.00
Overall OR (95% CI) §	0.80 (0.32–1.98)	0.59 (0.28–1.25) ^║^	1.00	0.49 (0.20–1.17) ^║^	0.80 (0.46–1.38)	1.00

* n = 31 participants did not have HCV RNA levels measured at a week 12 and were thus excluded from the EVR analysis

† OR (95% CI) adjusted for age (years), sex, IFNL4 genotype (ΔG/ΔG, ΔG/TT, TT/TT), BMI (kg/m^2^), albumin (g/dL), AST/ALT, platelet count (x10^3^/mm^3^), HOMA2 score, and baseline HCV RNA level (log_10_ transformed)

‡ OR (95% CI) adjusted for age (years), sex, IFNL4 genotype (ΔG/ΔG, ΔG/TT, TT/TT), BMI (kg/m^2^) AST/ALT, HOMA score, baseline HCV RNA level (log_10_ transformed), and treatment site

§ Overall multivariable ORs estimated from a random-effects meta-analysis

^║^ Significant study heterogeneity (I^2^>50%) for the pair of multivariable ORs

Abbreviations: 25(OH)D, 25-hydroxyvitamin D; AST/ALT, aspartate transaminase and alanine transaminase ratio; BMI, body mass index; CI, confidence interval; HCV, hepatitis C virus; HOMA, homeostasis model assessment; OR, odds ratio; PEG-IFNα/RBV, pegylated interferon alpha and ribavirin

Among African Americans, vitamin D status, defined using either IOM cut-points or a 20 ng/mL cutpoint, was not associated with virologic response (all P-values>0.05) (Table 4). In secondary analyses, we found similar results using season- and race-specific quartiles of vitamin D (S4 Table).

**Table 4:** Associations of baseline serum 25(OH)D status with response to PEG-IFNα/RBV treatment for HCV among African Americans.

	Baseline serum 25(OH)D concentrations (ng/mL)					
	IOM cut points				Meta-analysis cut points (31)	
	<12	12 to <20	20 to <30 (Ref)	≥30	**<20**	**≥20 (Ref)**
**Early virologic response**						
HALT-C, n responders/n total *	22/80	18/57	7/19	1/3	40/137	8/22
Multivariable adjusted OR (95% CI) †	0.52 (0.15–1.73)	0.63 (0.19–2.13)	1.00	0.87 (0.05–16.16)	0.58 (0.20–1.67)	1.00
VIRAHEP-C, n responders/n total	26/62	30/74	18/41	2/7	56/136	20/48
Multivariable adjusted OR (95% CI) ‡	0.91 (0.31–2.68)	0.64 (0.23–1.77)	1.00	0.84 (0.12–5.72)	0.77 (0.32–1.84)	1.00
Overall OR (95% CI) §	0.71 (0.32–1.59)	0.64 (0.29–1.39)	1.00	0.85 (0.17–4.22)	0.68 (0.35–1.34)	1.00
**Sustained virologic response**						
HALT-C, n responders/n total	8/84	2/60	1/19	0/3	10/144	1/22
Multivariable adjusted OR (95% CI) †	0.83 (0.07–9.48)	0.19 (0.01–3.39)	1.00	—	0.77(0.07–8.17)	1.00
VIRAHEP-C, n responders/n total	20/62	18/74	12/41	0/7	38/136	12/48
Multivariable adjusted OR (95% CI) ‡	0.96 (0.32–2.89)	0.45 (0.15–1.33)	1.00	—	0.82 (0.32–2.06)	1.00
Overall OR (95% CI) §	0.94 (0.34–2.56)	0.40 (0.14–1.11)	1.00	—	0.81 (0.34–1.92)	1.00

* n = 7 participants did not have HCV RNA levels measured at a week 12 and were thus excluded from the EVR analysis

† OR (95% CI) adjusted for age (years), sex, IFNL4 genotype (ΔG/ΔG, ΔG/TT, TT/TT), BMI (kg/m^2^), albumin (g/dL), AST/ALT, platelet count (x10^3^/mm^3^), HOMA2 score, and baseline HCV RNA level (log_10_ transformed)

‡ OR (95% CI) adjusted for age (years), sex, IFNL4 genotype (ΔG/ΔG, ΔG/TT, TT/TT), BMI (kg/m^2^) AST/ALT, HOMA score, baseline HCV RNA level (log_10_ transformed), and treatment site

§ Overall multivariable ORs estimated from a random-effects meta-analysis

Abbreviations: 25(OH)D, 25-hydroxyvitamin D; AST/ALT, aspartate transaminase and alanine transaminase ratio; BMI, body mass index; CI, confidence interval; HCV, hepatitis C virus; HOMA, homeostasis model assessment; OR, odds ratio; PEG-IFNα/RBV, pegylated interferon alpha and ribavirin

Similar associations were observed when we adjusted more comprehensively for markers of liver disease, in unadjusted models (data not shown) and in models that were stratified by severity of fibrosis. Excluding patients with cirrhosis (i.e. Ishak stage of 5 or 6) did not meaningfully alter the associations between vitamin D and virologic response (Table 5) among European Americans, although our statistical power was limited in VIRAHEP-C due to a smaller sample size. In season-stratified analyses, observed associations between high vitamin D status and worse response to treatment were stronger for samples collected in the summer but not the winter months (Table 5).

**Table 5:** Associations of baseline serum 25(OH)D status with response to HCV-treatment in the HALT-C and VIRAHEP-C studies stratified by Ishak stage and season among European Americans.

	Baseline serum 25(OH)D concentrations (ng/mL)		
	<20	20 to <30 (Ref)	≥30
**EVR**			
**Overall**			
HALT-C multivariable adjusted OR (95% CI) *	1.05 (0.72–1.52)	1.00	0.60 (0.38–0.95)
VIRAHEP-C multivariable adjusted OR (95% CI) †	0.27 (0.11–0.70)	1.00	0.76 (0.33–1.74)
**Ishak stage 1–4 (fibrosis)**			
HALT-C multivariable adjusted OR (95% CI) *	1.03 (0.64–1.66)	1.00	0.48 (0.27–0.83)
VIRAHEP-C multivariable adjusted OR (95% CI) †	0.31 (0.10–0.95)	1.00	0.66 (0.26–1.72)
**Ishak stage 5 or 6 (cirrhosis)**			
HALT-C multivariable adjusted OR (95% CI) *	1.12 (0.59–2.12)	1.00	0.84 (0.37–1.90)
VIRAHEP-C multivariable adjusted OR (95% CI) †	—	—	—
**Summer (June to November)**			
HALT-C multivariable adjusted OR (95% CI) * ‡	1.49 (0.81–2.76)	1.00	0.47 (0.26–0.83)
VIRAHEP-C multivariable adjusted OR (95% CI) †	0.14 (0.03–0.67)	1.00	0.44 (0.15–1.29)
**Winter (December to May)**			
HALT-C multivariable adjusted OR (95% CI) * ‡	0.83 (0.49–1.38)	1.00	1.27 (0.53–3.02)
VIRAHEP-C multivariable adjusted OR (95% CI) †	0.59 (0.13–2.69)	1.00	1.16 (0.15–9.01)
**SVR**			
**Overall**			
HALT-C multivariable adjusted OR (95% CI) *	0.74 (0.45–1.22)	1.00	0.31 (0.14–0.66)
VIRAHEP-C multivariable adjusted OR (95% CI) †	0.49 (0.22–1.11)	1.00	0.76 (0.37–1.57)
**Ishak stage 1–4 (fibrosis)**			
HALT-C multivariable adjusted OR (95% CI) *	0.83 (0.46–1.51)	1.00	0.33 (0.17–0.77)
VIRAHEP-C multivariable adjusted OR (95% CI) †	0.48 (0.18–1.23)	1.00	0.69 (0.31–1.54)
**Ishak stage 5 or 6 (cirrhosis)**			
HALT-C multivariable adjusted OR (95% CI) *	0.58 (0.22–1.55)	1.00	0.16 (0.02–1.38)
VIRAHEP-C multivariable adjusted OR (95% CI) †	—	—	—
**Summer (June to November)**			
HALT-C multivariable adjusted OR (95% CI) *	0.85 (0.38–1.90)	1.00	0.22 (0.09–0.57)
VIRAHEP-C multivariable adjusted OR (95% CI) †	0.45 (0.13–1.52)	1.00	0.69 (0.29–1.62)
**Winter (December to May)**			
HALT-C multivariable adjusted OR (95% CI) *	0.71 (0.36–1.40)	1.00	0.65 (0.15–2.84)
VIRAHEP-C multivariable adjusted OR (95% CI) †	0.90 (0.21–3.97)	1.00	0.63 (0.09–4.39)

* OR (95% CI) adjusted for age (years), sex, IFNL4 genotype (ΔG/ΔG, ΔG/TT, TT/TT), BMI (kg/m^2^), albumin (g/dL), AST/ALT, platelet count (x10^3^/mm^3^), HOMA2 score, and baseline HCV RNA level (log_10_ transformed)

† OR (95% CI) adjusted for age (years), sex, IFNL4 genotype (ΔG/ΔG, ΔG/TT, TT/TT), BMI (kg/m^2^) AST/ALT, HOMA score, baseline HCV RNA level (log_10_ transformed), and treatment site

‡ All P-value for heterogeneity < 0.05

Abbreviations: 25(OH)D, 25-hydroxyvitamin D; AST/ALT, aspartate transaminase and alanine transaminase ratio; BMI, body mass index; CI, confidence interval; EVR, early virologic response; HCV, hepatitis C virus; HOMA, homeostasis model assessment; IOM, Institute of Medicine; OR, odds ratio; SVR, sustained virologic response

**Fig 2:**
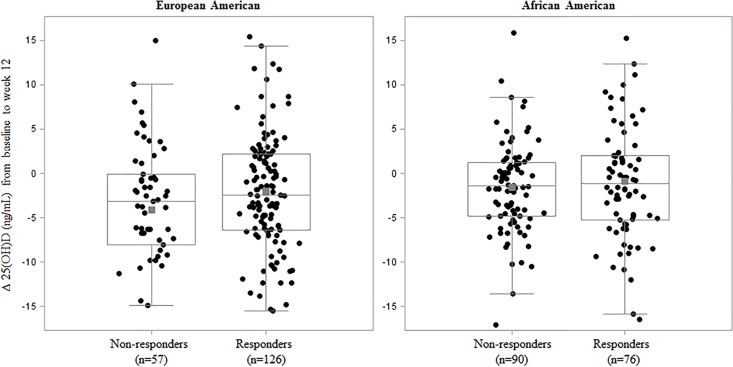
Race-specific distribution of change in vitamin D, 25(OH)D (ng/mL), from baseline to week 12 stratified by early virologic response status in the VIRAHEP-C study. Individual change in vitamin D was calculated as the change in serum 25(OH)D (ng/mL) at week 12 of treatment compared with baseline [i.e. 25(OH)D _week12_−25(OH)D _baseline_] and was adjusted for age (years), sex, IFNL4 genotype (ΔG/ΔG, ΔG/TT, TT/TT), BMI (kg/m^2^), AST/ALT, HOMA score, baseline HCV RNA level (log_10_ transformed IU/mL), and treatment site. These adjusted values are plotted as black circles. The grey square represents the adjusted sample mean, and the horizontal grey lines indicate the adjusted sample median and interquartile range.

Finally, we considered the longitudinal relationship between 25(OH)D and EVR in VIRAHEP-C subjects with paired baseline and week 12 serum samples. Overall, serum 25(OH)D concentration decreased by an average of 2.7 and 1.2 ng/mL from baseline to week 12 among European (P-value for paired t-test<0.001) and African (P-values for paired t-test = 0.009) Americans, respectively. As illustrated in Fig 2, decreases in 25(OH)D concentrations appeared greater among non-responders than responders; however, these differences were not statistically significant in European Americans (mean 25(OH)D change = -2.00 ± 7.26 ng/mL and -4.09 ± 7.97 ng/mL for early and non-responders, respectively; P value for unpaired t-test = 0.08) or in African Americans (mean 25(OH)D change = -0.85 ± 6.80 ng/mL and -1.53 ± 5.07 ng/mL for early and non-responders, respectively; P value for unpaired t-test = 0.47) (Fig 2).

## Discussion

We examined the association between vitamin D status, as measured by serum 25(OH)D, and response to PEG-IFNα/RBV treatment for chronic HCV genotype 1 infection in two large, high quality studies. In contrast to the hypothesis, our data do not provide evidence for an association between higher serum 25(OH)D and higher likelihood of response to treatment. If anything, the patients with higher vitamin D concentrations had a worse response. Furthermore, we saw no association between vitamin D status and response to treatment among African Americans, despite their lower measured concentrations of vitamin D.

Several previous studies have examined the association between vitamin D status and response to PEG-IFNα/RBV therapy; the results have, however, been inconsistent, as evidenced by two recently published and conflicting meta-analyses on the subject [30, 31]. The meta-analysis by Kitson et al. considered 2,605 patients from 11 studies and concluded that baseline vitamin D status was not associated with SVR [30]. Unlike Kitson et al., the meta-analysis by García-Álvarez et al. included studies involving patients co-infected with human immunodeficiency virus (HIV) but did not include published abstracts [31]. This meta-analysis included data from 1,406 patients from 11 studies, only two of which overlapped with Kitson et al. and three of which were related to the same Italian cohort of approximately 200 patients [45–48], and concluded that low vitamin D status (i.e. <20 ng/mL) was associated with lower odds (OR = 0.53, 95% CI = 0.31–0.91) of attaining SVR following PEG-IFNα/RBV therapy when HCV genotypes 1, 2, 3 and 4 were combined [31]. The authors later reported that this association was no longer significant after excluding two of the three Italian studies [49]. The discordant findings of these meta-analyses may also be due to differences in search methods [45] and definitions of vitamin D status. In the current analysis of data from HALT-C and VIRAHEP-C, we did not replicate the association observed by García-Álvarez et al. for low vitamin D status (i.e. <20 ng/mL) and SVR among European Americans (OR = 0.80, 95% CI = 0.46–1.38) or African Americans (OR = 0.81, 95% CI = 0.34–1.92).

Previous studies of the association between vitamin D status and SVR have varied considerably in their ability to adjust for potential confounders including known clinical and genetic predictors of virologic response to PEG-IFNα/RBV therapy as well as demographic and environmental determinants of vitamin D status [30, 31], and they often lacked extensive clinical data on underlying liver disease. A major strength of our study is the high quality patient data that was collected by both the HALT-C and VIRAHEP-C trials. Accordingly, we examined potential confounding by demographic factors (i.e. age and sex), *IFNL4* genotype, which was not available in previous studies, and a comprehensive suite of systematically measured clinical markers of liver disease. Moreover, only one previous study considered the association of vitamin D and SVR separately among African Americans [50]; this cross-sectional study by Weintraub et al., which included 106 African Americans and 65 whites who were chronically infected with HCV genotype 1, did not adjust for potential confounding factors and pretreatment serum was not available for all patients. Similar to our findings, Weintraub et al., found no evidence of an association between vitamin D status and SVR following PEG-IFNα/RBV therapy among African Americans. Contrary to our findings, Weintraub et al. reported that higher vitamin D status was associated with higher rates of SVR among white patients [50].

Our finding that high vitamin D status, ≥30 ng/mL, is associated with significantly lower odds of early virologic response to treatment, particularly for treatment that commenced during summer months, is unexpected but is also in line with a growing body of evidence that indicates that very high vitamin D concentrations may have unexpected and adverse health effects [32, 42]. The 2011 IOM report on calcium and vitamin D described potential U- or reverse J-shaped associations of vitamin D with all-cause mortality, several cancers, cardiovascular risk, falls and fractures [32]. One prior study of vitamin D status and SVR following PEG-IFNα/RBV therapy for chronic HCV genotype 1 infection reported significantly higher prevalence of vitamin D concentrations <30 ng/ among those who achieved SVR than those who did not [51]. At the same time, our finding is at odds with two small supplementation studies, neither of which was placebo controlled, that found evidence for higher rates SVR rates among those receiving vitamin D supplementation [22, 23]. Additionally, our data conflict with the observation that patients have higher vitamin D concentrations once they attain SVR than they did before therapy [24] as we found a general decline in vitamin D concentrations as well as HCV RNA levels from baseline to treatment week 12. Moreover, HCV RNA levels at baseline were not lower among those with higher vitamin D concentrations, as would be expected if vitamin D had a direct antiviral effect.

There are several potential explanations for our unexpected findings. First, unmeasured or poorly measured variables associated with higher vitamin D concentrations as well as lower odds of response to treatment may account for the observed association. Second, it could be a spurious finding owing to chance, although we note consistent findings for high vitamin D status in our two contributing studies. It is also possible to obtain varying results owing to the random draws obtained when imputing missing data; however, we observed similar results for analyses using indicator variables for missing data instead of imputation (data not shown). Finally, we speculate that very high rates of hydroxylation of vitamin D_2_ and D_3_ to 25(OH)D in the liver may reduce the effectiveness of PEG-IFNα/RBV therapy by disrupting the metabolic pathway of one or both drugs.

Our study had several limitations. First, our results are based on observational data and should therefore be interpreted with caution. Although serum samples were obtained at baseline prior to the start of study treatment, earlier interferon therapy in HALT-C, underlying liver disease, and unmeasured, poorly measured, or unknown confounders may have impacted baseline vitamin D status as well as subsequent response to therapy. Second, despite a large sample size of African Americans, we had limited statistical power to evaluate the association of high vitamin D status with response to PEG-IFNα/RBV therapy among this group of patients because less than 3% of African Americans in our study, as compared with 23% of European Americans, had 25(OH)D concentrations ≥ 30 ng/mL. We note however, that vitamin D concentrations are typically lower among African-Americans than European-Americans and so we would expect that only a very small proportion of African-Americans in the general population have vitamin D concentrations that are ≥ 30 ng/mL.

## Conclusions

In conclusion, our study found no evidence that low vitamin D status as compared with vitamin D sufficiency, defined by the IOM as serum 25(OH)D between 20 to 30 ng/mL, was associated with response to PEG-IFNα/RBV treatment for chronic HCV genotype 1 infection among African or European Americans. Our data did, however, indicate that high vitamin D status, ≥30 ng/mL, was associated with lower odds of early virologic response among European Americans; however, this unexpected finding could be due to chance and should be interpreted with caution. While this study cannot comment directly on the efficacy of vitamin D supplementation as adjuvant for PEG-IFNα/RBV therapy, our data do not support the hypothesis that high pre-treatment vitamin D levels favorably impact response to HCV treatment.

## Supporting Information

S1 File NIH Publishing Agreement & Manuscript Cover Sheet. (PDF)

S1 Table Baseline characteristics by serum vitamin D status in the HALT-C study (N = 911). (DOCX)

S2 Table Baseline characteristics by serum vitamin D status in the VIRAHEP-C study (N = 381). (DOCX)

S3 Table Mean ΔHCV RNA level from baseline to day 28 of PEG-IFNα/RBV treatment‡ stratified by baseline serum 25(OH)D status and race in the VIRAHEP-C study. (DOCX)

S4 Table Associations of race- and season-specific quartile of serum vitamin D, 25(OH)D, with EVR and SVR in the HALT-C and VIRAHEP-C studies. (DOCX)
