# Supplementary material for: Vitamin D Status and Virologic Response to HCV Therapy in the HALT-C and VIRAHEP-C Trials
Source: PLoS One. 2016 Nov 10;11(11):e0166036. doi: 10.1371/journal.pone.0166036 (PMC5104464; doi:10.1371/journal.pone.0166036)
Supplement: S1 Table — (DOCX) [file pone.0166036.s002.docx]

**S1 Table. Baseline characteristics by serum vitamin D status in the HALT-C study (N=911).**

|  | **Baseline serum 25(OH)D concentrations (ng/mL)** | | | |  |
| --- | --- | --- | --- | --- | --- |
| **Baseline characteristic** | **<12** | **12 to <20** | **20 to <30 (Ref)** | **≥30** | **P*** |
| n (%) | 157 (17.2) | 281 (30.9) | 325 (35.7) | 148 (16.3) | --- |
| Age (y), median (IQR) | 50 (47-54) | 50 (46-54) | 49 (45-53) | 48 (45-52) | 0.0004 |
| Sex, n (%) |  |  |  |  | <0.0001 |
| Male | 92 (13.9) | 195 (29.6) | 267 (40.5) | 106 (16.1) |  |
| Female | 65 (25.9) | 86 (34.3) | 58 (23.1) | 42 (16.7) |  |
| Race, n (%) |  |  |  |  | <0.0001 |
| African American | 84 (50.6) | 60 (36.1) | 19 (11.5) | 3 (1.8) |  |
| European American | 73 (9.8) | 221 (29.7) | 306 (41.1) | 145 (19.5) |  |
| *IFNL4* (rs368234815) genotype, n (%)† |  |  |  |  | 0.003 |
| ΔG/ΔG | 48 (24.6) | 59 (30.3) | 56 (28.7) | 32 (16.4) |  |
| ΔG/TT | 69 (15.4) | 134 (29.9) | 164 (36.6) | 81 (18.1) |  |
| TT/TT | 18 (10.5) | 55 (32.2) | 76 (44.4) | 22 (12.9) |  |
| Ishak fibrosis stage, n (%) |  |  |  |  | 0.140 |
| ≤2 | 11 (15.1) | 21 (28.8) | 28 (38.4) | 13 (17.8) |  |
| 3 or 4 | 74 (14.7) | 155 (30.7) | 186 (36.8) | 90 (17.8) |  |
| 5 or 6 | 72 (21.7) | 105 (31.6) | 111 (33.4) | 44 (13.3) |  |
| BMI (kg/m^2^), n (%) |  |  |  |  | 0.001 |
| 18.5 to <25 | 25 (15.8) | 49 (31.0) | 47 (29.8) | 37 (23.4) |  |
| 25 to <30 | 58 (15.7) | 98 (26.6) | 143 (38.8) | 70 (19.0) |  |
| ≥30 | 74 (19.3) | 134 (34.9) | 135 (35.2) | 41 (10.7) |  |
| Season of baseline blood draw, n (%) |  |  |  |  | <0.0001 |
| Summer | 34 (7.5) | 116 (25.7) | 186 (41.2) | 116 (25.7) |  |
| Winter | 123 (26.8) | 165 (36.0) | 139 (30.3) | 32 (7.0) |  |
| HCV RNA level log_10_ (IU/mL), median (IQR) | 6.4 (6.1-6.8) | 6.5 (6.1-6.8) | 6.6 (6.2-6.8) | 6.5 (6.2-6.8) | 0.114 |
| Albumin (g/dL), median (IQR) | 3.8 (3.5-4.0) | 3.9 (3.6-4.2) | 4.0 (3.7-4.2) | 3.9 (3.7-4.2) | <0.0001 |
| AST/ALT, median (IQR) | 0.9 (0.7-1.1) | 0.8 (0.7-1.0) | 0.7 (0.6-0.9) | 0.8 (0.6-0.9) | <0.0001 |
| Alkaline phosphatase ratio (ULN), median (IQR) | 0.9 (0.7-1.1) | 0.8 (0.6-1.0) | 0.7 (0.6-0.9) | 0.7 (0.5-0.8) | <0.0001 |
| Bilirubin (mg/dL), median (IQR) | 0.7 (0.5-1.0) | 0.7 (0.5-1.0) | 0.7 (0.5-0.9) | 0.7 (0.5-0.9) | 0.318 |
| Platelet count (x10^3^/mm^3^), median (IQR) | 154 (112-201) | 163 (117-212) | 172 (132-216) | 175 (131-207) | 0.011 |
| HOMA2 score, median (IQR) | 9.5 (6.6-17.2) | 9.0 (5.2-15.4) | 7.3 (4.5-14.8) | 6.8 (4.4-12.7) | <0.0001 |

*P for Cochran-Armitage trend test for categorical variables (with 2 categories) and Jonckhere-Terpstra trend test for continuous variables; X^2^ test for categorical variables with >2 categories

†n=814 due to missing data

Abbreviations: 25(OH)D, 25-hydroxyvitamin D; AST/ALT, aspartate transaminase and alanine transaminase ratio; BMI, body mass index; HCV, hepatitis C virus; HOMA, homeostasis model assessment; IQR, interquartile range
