# Supplementary material for: Vitamin D Status and Virologic Response to HCV Therapy in the HALT-C and VIRAHEP-C Trials
Source: PLoS One. 2016 Nov 10;11(11):e0166036. doi: 10.1371/journal.pone.0166036 (PMC5104464; doi:10.1371/journal.pone.0166036)
Supplement: S2 Table — (DOCX) [file pone.0166036.s003.docx]

**S2 Table. Baseline characteristics by serum vitamin D status in the VIRAHEP-C study (N=381)**

|  | **Baseline serum 25(OH)D concentrations (ng/mL)** | | | |  |
| --- | --- | --- | --- | --- | --- |
| **Baseline characteristic** | **<12** | **12 to <20** | **20 to <30** | **≥30** | **P*** |
| n (%) | 74 (19.4) | 110 (28.9) | 120 (31.5) | 77 (20.2) | --- |
| Age (y), median (IQR) | 49 (45-53) | 48 (43-53) | 49 (44-53) | 48 (43-53) | 0.660 |
| Sex, n (%) |  |  |  |  | 0.019 |
| Male | 41 (16.6) | 69 (27.9) | 81 (32.8) | 56 (22.7) |  |
| Female | 33 (24.6) | 41 (30.6) | 39 (29.1) | 21 (15.7) |  |
| Race, n (%) |  |  |  |  | <0.0001 |
| African American | 62 (33.7) | 74 (40.2) | 41 (22.3) | 7 (3.8) |  |
| European American | 12 (6.1) | 36 (18.3) | 79 (40.1) | 70 (35.5) |  |
| *IFNL4* (rs368234815) genotype, n (%) |  |  |  |  | 0.0004 |
| ΔG/ΔG | 29 (32.6) | 23 (25.8) | 23 (25.8) | 14 (15.7) |  |
| ΔG/TT | 32 (12.1) | 45 (29.6) | 41 (27.0) | 34 (22.4) |  |
| TT/TT | 6 (6.5) | 23 (25.0) | 41 (44.6) | 22 (23.9) |  |
| Ishak fibrosis stage, n (%) |  |  |  |  | 0.701 |
| ≤2 | 50 (20.8) | 68 (28.3) | 70 (29.2) | 52 (21.7) |  |
| 3 or 4 | 18 (16.2) | 31 (27.9) | 40 (36.0) | 22 (19.8) |  |
| 5 or 6 | 5 (18.5) | 9 (33.3) | 10 (37.0) | 3 (11.1) |  |
| BMI (kg/m^2^), n (%) |  |  |  |  | 0.002 |
| 18.5 to <25 | 14 (15.1) | 22 (23.7) | 35 (37.6) | 22 (23.7) |  |
| 25 to <30 | 22 (15.9) | 44 (31.9) | 33 (23.9) | 39 (28.3) |  |
| ≥30 | 37 (25.5) | 42 (29.0) | 50 (34.5) | 16 (11.0) |  |
| Season of baseline blood draw, n (%) |  |  |  |  | <0.0001 |
| Summer | 32 (13.9) | 56 (24.4) | 80 (34.8) | 62 (27.0) |  |
| Winter | 42 (27.8) | 54 (35.8) | 40 (26.5) | 15 (9.9) |  |
| HCV RNA level log_10_ (IU/mL), median (IQR) | 6.3 (5.5-6.6) | 6.5 (5.6-6.8) | 6.5 (6.0-6.7) | 6.6 (6.1-6.8) | 0.006 |
| Albumin (g/dL), median (IQR) | 4.0 (3.6-4.2) | 4.0 (3.9-4.3) | 4.2 (3.9-4.4) | 4.2 (4.1-4.4) | <0.0001 |
| AST/ALT, median (IQR) | 0.9 (0.7-1.1) | 0.8 (0.6-1.0) | 0.7 (0.6-0.9) | 0.7 (0.6-0.8) | <0.0001 |
| Alkaline phosphatase (U/L), median (IQR) | 87 (69-113) | 80 (62-106) | 76 (59-95) | 75 (62-92) | 0.002 |
| Bilirubin (mg/dL), median (IQR) | 0.6 (0.4-0.8) | 0.6 (0.4-0.8) | 0.7 (0.5-0.9) | 0.7 (0.5-0.9) | 0.034 |
| Platelet count (x10^3^/mm^3^), median (IQR) | 215 (161-268) | 214 (164-262) | 204 (163-251) | 197 (164-240) | 0.213 |
| HOMA score, median (IQR) | 3.3 (2.0-5.9) | 3.8 (2.0-7.7) | 2.7 (1.7-5.5) | 2.6 (1.4-3.9) | 0.001 |

*P for Cochran-Armitage trend test for categorical variables (with 2 categories) and Jonckhere-Terpstra trend test for continuous variables; X^2^ test for categorical variables with >2 categories

Abbreviations: 25(OH)D, 25-hydroxyvitamin D; AST/ALT, aspartate transaminase and alanine transaminase ratio; BMI, body mass index; HCV, hepatitis C virus; HOMA, homeostasis model assessment; IOM, Institute of Medicine; IQR, interquartile rang
