# Supplementary material for: Vitamin D Status and Virologic Response to HCV Therapy in the HALT-C and VIRAHEP-C Trials
Source: PLoS One. 2016 Nov 10;11(11):e0166036. doi: 10.1371/journal.pone.0166036 (PMC5104464; doi:10.1371/journal.pone.0166036)
Supplement: S3 Table — (DOCX) [file pone.0166036.s004.docx]

**S3 Table. Mean ΔHCV RNA level from baseline to day 28 of PEG-IFNα/RBV treatment‡ stratified by baseline serum 25(OH)D status and race in the VIRAHEP-C study.**

|  | **Baseline serum 25(OH)D concentrations (ng/mL)** | | | |
| --- | --- | --- | --- | --- |
|  | **<12** | **12 to <20** | **20 to <30** | **≥30** |
| European Americans |  |  |  |  |
| n | 10 | 35 | 77 | 69 |
| Crude LS mean (SE) | -2.20 (0.38) | -1.71 (0.20) | -2.50 (0.14) | -2.12 (0.14) |
| Minimally-adjusted LS mean (SE)* | -2.45 (0.40) | -1.49 (0.19) | -2.18 (0.13) | -1.95 (0.14) |
| Fully-adjusted LS mean (SE)† | -2.35 (0.38) | -1.30 (0.19) | -2.13 (0.14) | -1.88 (0.16) |
| African Americans |  |  |  |  |
| n | 59 | 70 | 40 | 7 |
| Crude LS mean (SE) | -1.51 (0.13) | -1.55 (0.12) | -1.43 (0.16) | -1.30 (0.38) |
| Minimally-adjusted LS mean (SE)* | -1.85 (0.15) | -1.73 (0.14) | -1.72 (0.17) | -1.78 (0.35) |
| Fully-adjusted LS mean (SE)† | -1.67 (0.17) | -1.64 (0.16) | -1.56 (0.18) | -1.66 (0.36) |

***** Adjusted for age (years), sex, IFNL4 genotype (ΔG/ΔG, ΔG/TT, TT/TT), BMI (kg/m^2^), baseline HCV RNA level (log_10_ transformed IU/mL), HOMA score, treatment site, and AST/ALT

† Adjusted for age (years), sex, IFNL4 genotype (ΔG/ΔG, ΔG/TT, TT/TT), BMI (kg/m^2^), baseline HCV RNA level (log_10_ transformed IU/mL), HOMA score, treatment site, AST/ALT, albumin (g/dL), alkaline phosphatase (U/L), total bilirubin (mg/dL), platelet count (x10^3^/mm^3^), and Ishak stage (1 to 6)

‡ Calculated as the change in log_10_ transformed viral load from baseline to day 28 (*i.e. log_10_(HCV RNA) _day 28_ –log_10_(HCV RNA) _baseline_*)

Abbreviations: 25(OH)D, 25-hydroxyvitamin D; AST/ALT, aspartate transaminase and alanine transaminase ratio; BMI, body mass index; HCV, hepatitis C virus; HOMA, homeostasis model assessment; IOM, Institute of Medicine; LS, least squares; PEG-IFNα/RBV, pegylated interferon alpha and ribavirin; SE, standard error
