# Supplementary material for: Vitamin D Status and Virologic Response to HCV Therapy in the HALT-C and VIRAHEP-C Trials
Source: PLoS One. 2016 Nov 10;11(11):e0166036. doi: 10.1371/journal.pone.0166036 (PMC5104464; doi:10.1371/journal.pone.0166036)
Supplement: S4 Table — (DOCX) [file pone.0166036.s005.docx]

**S4 Table.** Associations of race- and season-specific quartile of serum vitamin D, 25(OH)D, with EVR and SVR in the HALT-C and VIRAHEP-C studies.

|  | **Race- and season-specific quartiles of serum 25(OH)D (ng/mL)** | | | |
| --- | --- | --- | --- | --- |
|  | **Q1 (Ref)** | **Q2** | **Q3** | **Q4** |
| European American, summer | <19.6 | 19.6 to <25.3 | 25.3 to <31.5 | ≥31.5 |
| European American, winter | <14.3 | 14.3 to <19.2 | 19.2 to <25.2 | ≥25.2 |
| African American, summer | <12.0 | 12.0 to <15.0 | 15.0 to <20.0 | ≥20.0 |
| African American, winter | <7.3 | 7.3 to <10.0 | 10.0 to <13.1 | ≥13.1 |
| **EVR** |  |  |  |  |
| HALT-C |  |  |  |  |
| n (% total) | 103 (11.8) | 109 (12.5) | 111 (12.7) | 98 (11.2) |
| Crude OR (95% CI) | 1.00 | 1.10 (0.75-1.60) | 1.10 (0.75-1.60) | 0.85 (0.58-1.23) |
| Multivariable adjusted OR (95% CI) * | 1.00 | 0.77 (0.49-1.20) | 0.74 (0.48-1.15) | 0.67 (0.43-1.04) |
| VIRAHEP-C |  |  |  |  |
| n (% total) | 48 (12.6) | 54 (14.2) | 55 (14.4) | 45 (11.8) |
| Crude OR (95% CI) | 1.00 | 1.23 (0.70-2.18) | 1.26 (0.71-2.22) | 0.88 (0.50-1.56) |
| Multivariable adjusted OR (95% CI) † | 1.00 | 1.11 (0.55-2.24) | 0.86 (0.42-1.75) | 0.84 (0.40-1.79) |
| **SVR** |  |  |  |  |
| HALT-C |  |  |  |  |
| n (% total) | 29 (3.2) | 32 (3.5) | 41 (4.5) | 19 (2.1) |
| Crude OR (95% CI) | 1.00 | 1.12 (0.65-1.91) | 1.49 (0.89-2.49) | 0.62 (0.34-1.15) |
| Multivariable adjusted OR (95% CI) * | 1.00 | 0.85 (0.45-1.58) | 1.04 (0.57-1.90) | 0.48 (0.24-0.95) |
| VIRAHEP-C |  |  |  |  |
| n (% total) | 35 (9.2) | 43 (11.3) | 39 (10.2) | 32 (8.4) |
| Crude OR (95% CI) | 1.00 | 1.39 (0.77-2.44) | 1.13 (0.63-2.03) | 0.87 (0.48-1.58) |
| Multivariable adjusted OR (95% CI) † | 1.00 | 1.20 (0.60-2.38) | 0.86 (0.43-1.71) | 0.83 (0.40-1.72) |

* OR (95% CI) adjusted for age (years), sex, IFNL4 genotype (ΔG/ΔG, ΔG/TT, TT/TT), Ishak stage (1 to 6), BMI (kg/m^2^), albumin (g/dL), AST/ALT, alkaline phosphatase ratio, total bilirubin (mg/dL), platelet count (x10^3^/mm^3^), HOMA2 score, and baseline HCV RNA level (log_10_ transformed)

† Adjusted for age (years), sex, IFNL4 genotype (ΔG/ΔG, ΔG/TT, TT/TT), BMI (kg/m^2^), baseline HCV RNA level (log_10_ transformed IU/mL), HOMA score, treatment site, AST/ALT, albumin (g/dL), alkaline phosphatase (U/L), total bilirubin (mg/dL), platelet count (x10^3^/mm^3^), and Ishak stage (1 to 6)

† OR (95% CI) adjusted for age (years), sex, IFNL4 genotype (ΔG/ΔG, ΔG/TT, TT/TT), Ishak stage (1 to 6), BMI (kg/m^2^) albumin (g/dL), AST/ALT, alkaline phosphatase (U/L), total bilirubin (mg/dL), platelet count (x10^3^/mm^3^), HOMA score, baseline HCV RNA level (log_10_ transformed), and treatment site

Abbreviations: BMI, body mass index; CI, confidence interval; EVR, early virologic response; HCV, hepatitis C virus; IOM, Institute of Medicine; OR, odds ratio; SVR, sustained virologic response
